# Adhesive Leaf Created by a Corona Discharge

**DOI:** 10.1038/s41598-018-19328-8

**Published:** 2018-01-29

**Authors:** Wonseok Lee, Jongsang Son, Seonghyun Kim, Dongmin Yang, Seungyeop Choi, Rodrigo Akira Watanabe, Kyo Seon Hwang, Sang Woo Lee, Gyudo Lee, Dae Sung Yoon

**Affiliations:** 1 Department of Biomedical Engineering, Yonsei University, Wonju, 26493 South Korea; 2 Shirley Ryan AbilityLab, Chicago, IL 60611 USA; 3 Department of Physical Medicine and Rehabilitation, Northwestern University, Chicago, IL 60611 USA; 4 School of Biomedical Engineering, Korea University, Seoul, 02841 South Korea; 5 School of Medicine, University of São Paulo, São Paulo, Brazil; 6 Department of Clinical Pharmacology and Therapeutics, College of Medicine, Kyung Hee University, Seoul, 02447 Korea

## Abstract

Here, we report a new concept of both the adhesive manner and material, named “adhesive leaf (AL),” based on the leaf of the plant *Heteropanax fragrans*. The treatment of the corona discharge on the leaf surface can cause the nano-/microdestruction of the leaf epidermis, resulting in an outward release of sap. The glucose-containing sap provided the AL with a unique ability to stick to various substrates such as steel, polypropylene, and glass. Moreover, we reveal that the AL adhesion strength depends on the AL size, as well as the corona-discharge intensity. Conventional adhesives, such as glue and bond, lose their adhesive property and leave dirty residues upon the removal of the attached material. Unlike the conventional methods, the AL is advantageous as it can be repeatedly attached and detached thoroughly until the sap liquid is exhausted; its adhesive ability is maintained for at least three weeks at room temperature. Our findings shed light on a new concept of a biodegradable adhesive material that is created by a simple surface treatment.

## Introduction

Evolutionary processes have created the diverse adhesive methods of the organisms in nature^1–6^. For instance, on each footpad of the gecko animal, hundreds of thousands of setae with a density of 5300/m^2^ exist^7^. Each seta consists of hundreds of spatulas, and each microspatula applies approximately 20 μN using a number of forces including the van der Waals, dipole, and capillary forces^1,2^. All of the forces are congregated and contribute to the adhesion of the gecko onto walls. In contrast to the manner of the gecko foot, chemical adhesive methods are also used in nature; for example, mussels can attach to all surfaces^4,6^. Mussels produce hair-like fibers that are composed of amino acids including 3,4-dihydrxy-L-phenylalanine and lysine, and the fibers allow the mussels to adhere to sea-rock surfaces. Another interesting organism, the *Onychophora*, squeezes out a slime known as a *food web* that overcomes its slow migration speed to obtain feed (prey); an adhesive property of the slime is enough to entangle the organism’s prey^8^.

Taking advantage of nature-developed techniques, the ancient Egyptians made paper from papyrus, which possesses an adhesive property without any additives^9^; since then, it appears that researchers have continuously studied the adhesive method of organisms for many centuries^1–3,5–7^. Today, this research area is called *biomimetics*^10^. By using the morphological benefit of the gecko footpad, for example, gecko tape has been developed to ensure a collective adhesion; a method of the microfabrication of dense arrays of flexible plastic pillars is used to make the gecko tape^7^. A mimicking of the high-strength adhesive material that is produced by mussels has shown that it is nontoxic to living cells, thereby suggesting its potential suitability for surgical and other biomedical applications^11^. Besides, many studies are currently underway to mimic the techniques in nature effectively in terms of biocompatible applications.

Meanwhile, the corona-discharge is generated by the application of a high voltage at high frequency to an electrode tip. The corona-treated surface becomes stiff, whereby often induces physical damage (surface degradation) of insulating materials (*e.g*., rubber)^12^. When the corona-discharge is exerted, the materials are discharged through the circumjacent fluid such as water and air. Corona-discharge treatment has been widely used to activate the surface of materials using high energy levels^13–15^. For instance, corona-discharged polydimethylsiloxane can strongly adhere to other materials such as glass, plastic, or itself without the additional use of adhesives^16,17^. In the present study, a report is presented on the leaves of *Heteropanax fragrans* for the attainment of an adhesive property as a corona discharge is treated. The treatment of the corona discharge changes not only the leaf epidermal structure, but it also alters the leaf adhesive functionality, thereby creating the *adhesive leaf (AL)*.

The corresponding mechanism is as follows: As the leaves are treated by the corona discharge, sap oozes from the leaves through the damaged epidermis, which is a phenomenon that is similar to a snail’s secretion of its adhesive mucus throughout its body^18^. Snail mucus plays a central role in the attachment regarding various surfaces as well as self-defense. Organic-compound mixtures such as polysaccharides, the proteins in the snail mucus, provide snails with a surface-attachment ability. It is confirmed here that the AL adhesion strength is augmented as the AL surface area is increased. Moreover, tests of the AL adhesive ability for various substrates such as steel, polymer, and glass were conducted. It is believed that the AL represents a novel concept of a biodegradable and eco-friendly adhesive material.

## Results and Discussion

**Figure 1:**
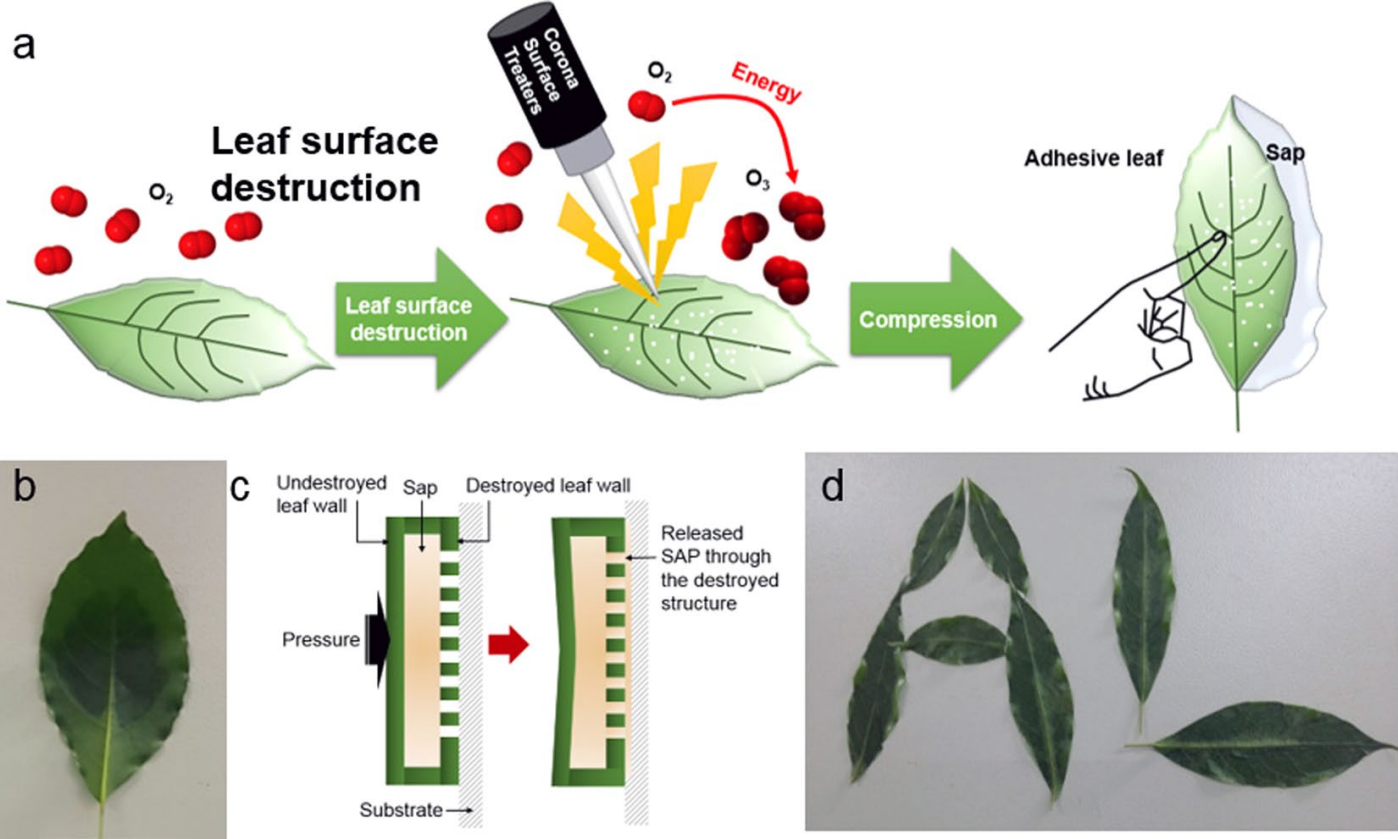
**(a)** Schematic illustration of the preparation of AL using a corona discharge. **(b)** An example of AL attached to the wall. **(c)** Schematic of the adhesive mechanism of AL with a side view. **(d)** AL patterning with different alphabets, ‘A’ and ‘L’.

When the corona discharge was applied to a *Heteropanax fragrans* leaf, the leaf adhered to the wall surfaces, as shown in Fig. 1(a) and (b); accordingly, the “adhesive leaf” name was derived. Because of the liquid leakage from the AL surface, it was predicted that the AL adhesive force is due to the fine sap flow through the micropores that is formed by the corona-discharge destruction of the leaf epidermal structure, as shown in Fig. 1(c). To verify whether this prediction is valid, a needle was used to puncture holes with diameters of a few millimeters into the leaves; however, the sap did not leak out due to its viscosity^19^, and the punctured leaves did not display an adhesive property (data not shown). In contrast, it was confirmed that the corona-discharge-created ALs could attach to the walls due to the adherence of the sap to the leaves, and the ALs were even patterned with alphabets, as shown in Fig. 1(d). In addition, the AL adhesive strength can persist until the sap is exhausted. Note that the number of attachments/detachments is dependent on the AL size and the quantity of sap inside the leaf; this will be discussed later. Even if the AL on the wall is dried, the adhesive ability can be maintained for at least three weeks. These results imply that the AL adhesive force is attributed to the flow of the sap through the micropores that are formed by the corona-induced destruction of the leaf epidermal structure.

**Figure 2:**
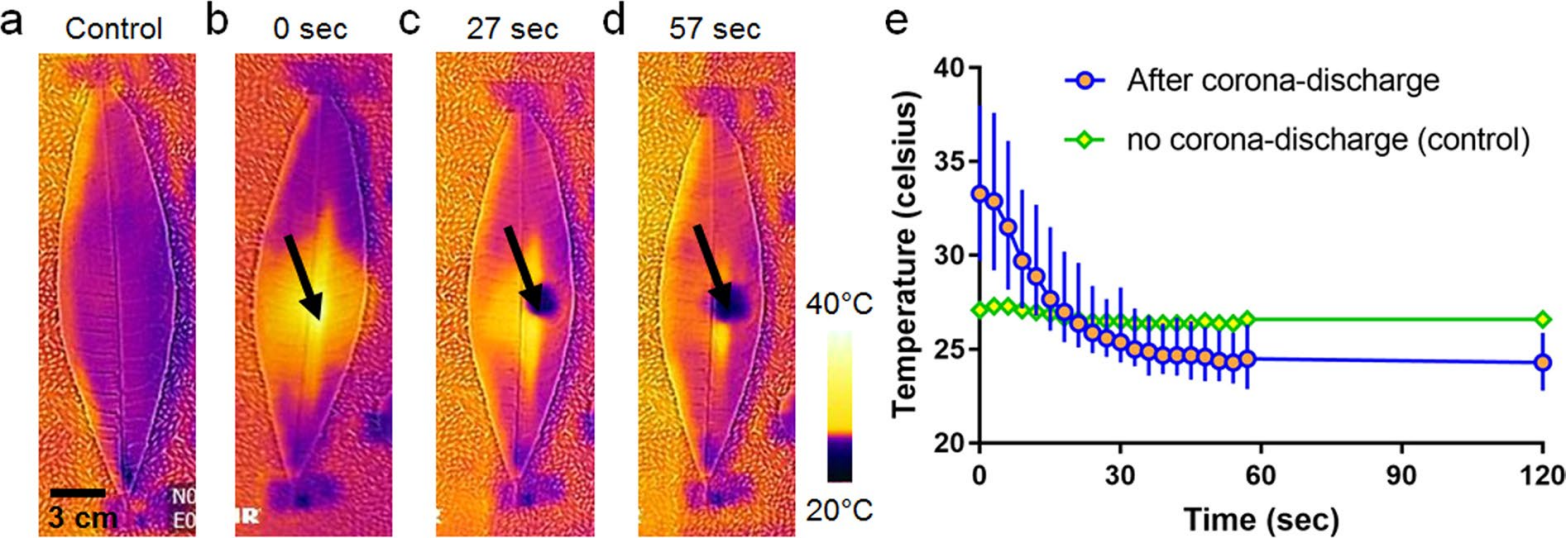
**(a–d)** Time-lapse thermal imaging of AL after corona treatment for about 120 s. Black arrows indicate corona treated spot of AL. **(e)** A temperature change of AL after corona treatment, which is compared with a leaf without corona treatment. Each data point represents triplicate measurements.

FTIR experiments were conducted to analyze the sap components that are the result of the corona treatment, as shown in Fig. S2. The subtraction of the sap wavelength band from the deionized (DI)-water wavelength band revealed that glucose is present in the sap^20^. The leakage of the sap from the AL was confirmed by the temperature changes in the corona-treated spots. Using an infrared camera, direct measurements of the temperature changes on the leaf surface before and after the corona discharge were performed. Prior to the corona treatment, the mean temperature of the leaf surface was measured as approximately 27 °C, as can be seen in Fig. 2(a). Immediately after the corona treatment, the temperature of a corona-treated spot on the leaf surface rose sharply up to approximately 33 °C, as shown in Fig. 2(b). After approximately 20 s, the spot temperature gradually decreased to a temperature that is even slightly lower than the pre-corona-treatment measurement, as shown in Fig. 2(c) and (d). The lowered temperature remained unchanged for at least 120 s, and this is evident in Fig. 2(e). As represented in Fig. S3, the temperature curve obeys the Boltzmann sigmoid model. These temperature changes imply that the corona treatment induced a highly thermal energy to destruct the leaf surface, and the sap flowed out from the damaged structures and was evaporated upon its contact with air. It was subsequently speculated that the AL corona-treated spots would show a lower temperature than those of the pristine leaves.

**Figure 3:**
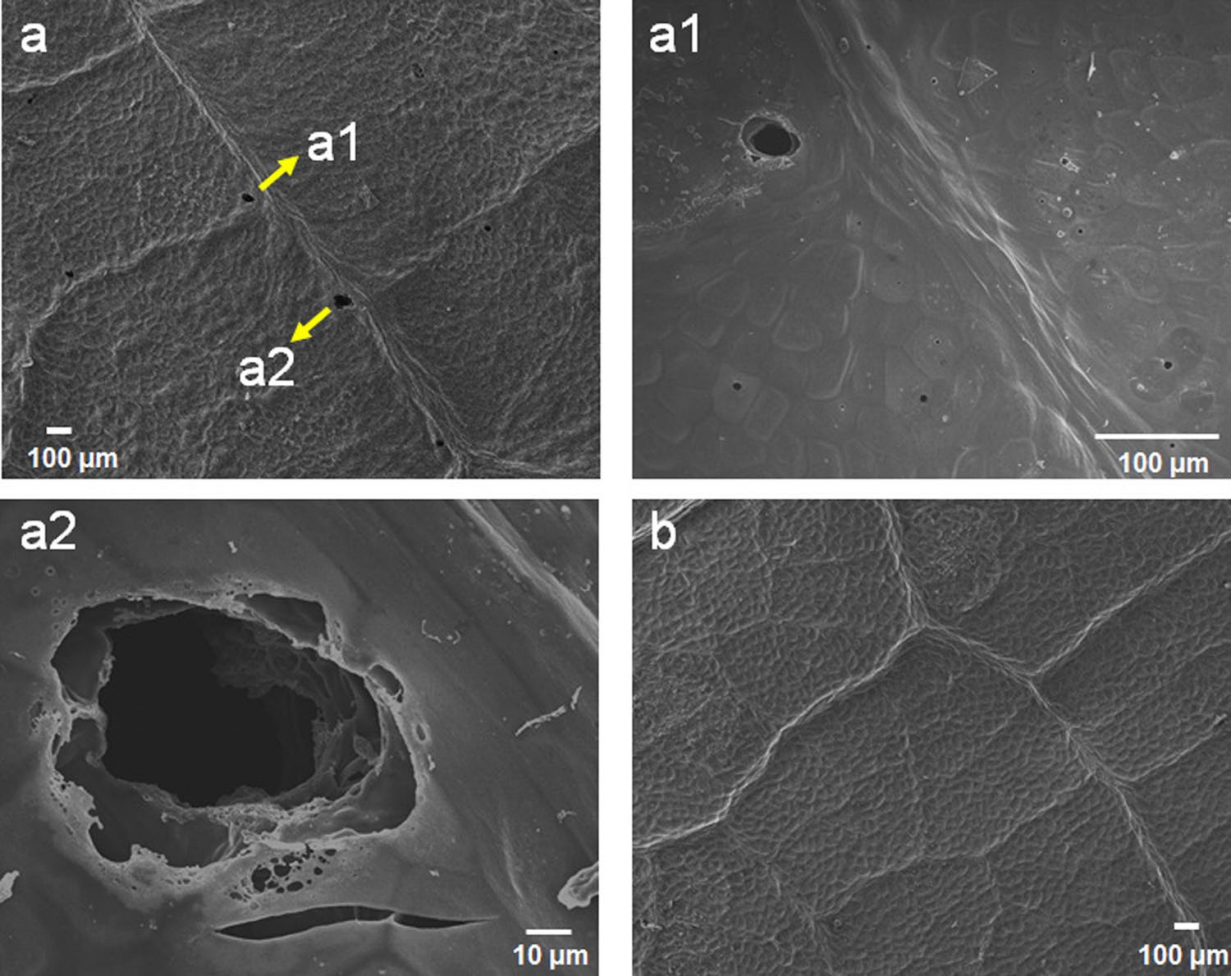
**(a)** Scanning electron microscopy (SEM) image of AL surface after corona treatment. (a1,a2) Magnified regions of AL from (**a**). **(b)** Negative control: SEM image of the surface of a normal leaf before (or without) corona treatment. Because the upper epidermis was analyzed by SEM, no stomata were observed.

**Figure 4:**
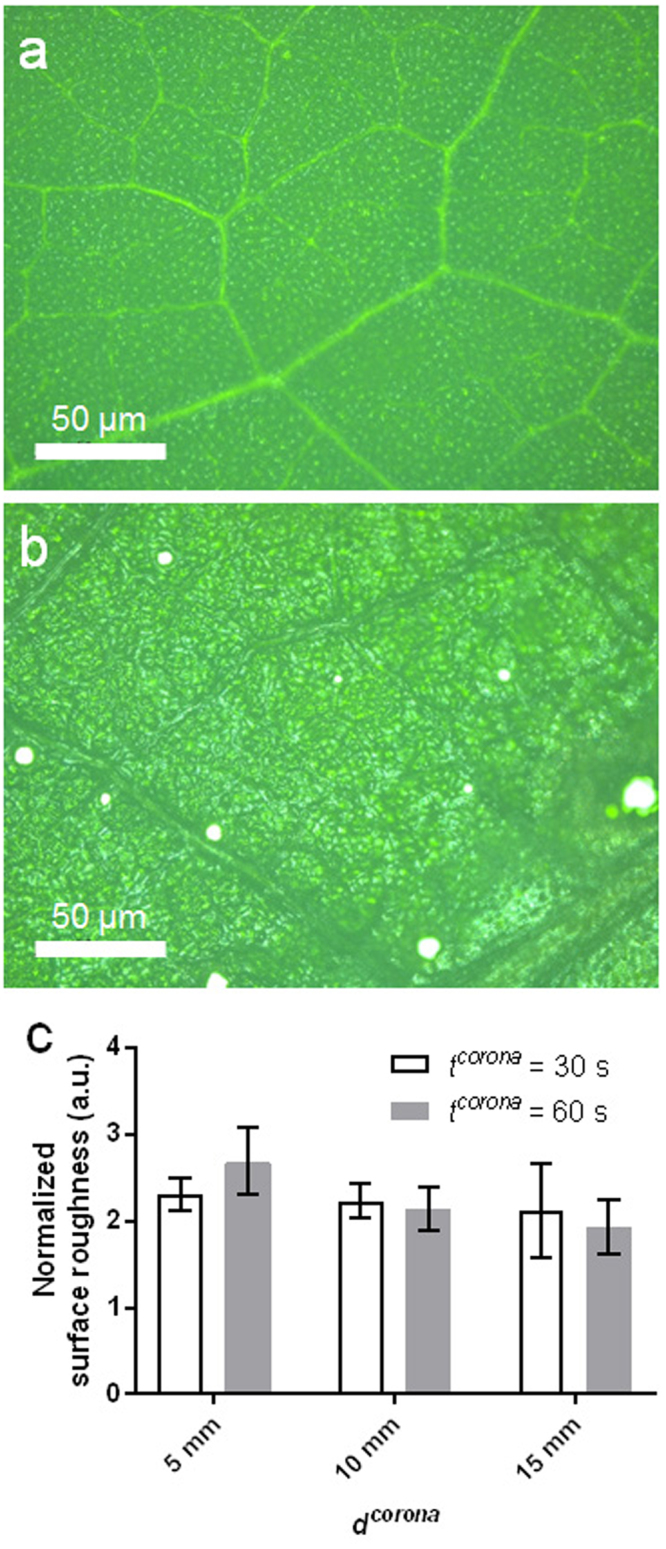
**(a,b)** Optical images of AL surface before (**a**) and after (**b**) corona treatment. **(c)** The normalized surface roughness change with using different *d*^*corona*^s (5–15 mm) and *t*^*corona*^s (30 and 60 s). The normalized surface roughness was calculated by the surface roughness of AL divided by the surface roughness of pristine leaf (before corona treatment). Each data point represents triplicate measurements.

An scanning electron microscopy (SEM) analysis of an AL corona-treated spot was performed to examine the damaged structures. The SEM images revealed that the corona discharge could create micropore structures (tens-of-μm sizes) on the AL surfaces compared with the pristine-leaf surfaces (control), and this can be seen in Fig. 3(a) and (b). The AL micropore structures were randomly created on the corona-treated spots, which may have played a role in the sap-leakage pathways contained inside the leaves. Similarly, the leaf surfaces before and after the corona treatment were observed using an optical microscope. Microsize pores became apparent as white dots after the corona treatment, and these are shown in Fig. 4(a) and (b). The white color of the dots may be due to the sap light reflection. Moreover, the AL surface, except for the white-dot structure, became very deteriorated compared with the pristine-leaf surface. A quantification of the degree of the damaged leaf surface was calculated using the normalized surface roughness with a different corona energy. To calculate the normalized surface roughness, the AL surface roughness was divided by the surface roughness of the pristine surface (before the corona treatment).

Also, the corona energy depends on either the distance (*d*^*corona*^) between the tip of a corona discharge and the sample (leaves) or between the tip of a corona discharge and the treatment time (*t*^*corona*^)^15^. Three different *d*^*corona*^ values of 5, 10 and 15 mm and two different *t*^*corona*^ values of 30 and 60 s were considered. The results show that the surface roughness was increased as the *d*^*corona*^ became closer and the *t*^*corona*^ was increased, as shown in Fig. 4(c). Interestingly, these results imply that the *d*^*corona*^ of 5 mm and the *t*^*corona*^ of 60 s are more effective for the formation of the damaged structures including the micropores compared with the other conditions.

**Figure 5:**
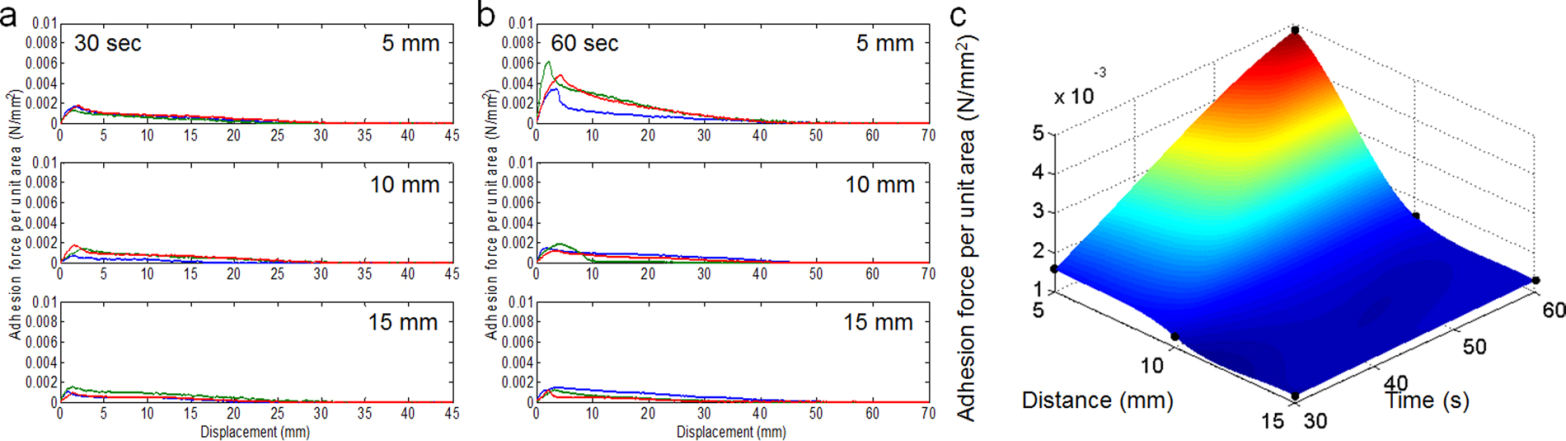
Adhesive forces per unit area-displacement curves obtained from the corona discharge time of 30 s **(a)** and 60 s **(b)**. **(c)** Peak adhesive forces at different corona discharging distance (i.e., 5, 10, and 15 mm) and time. Note that the adhesive force increases with closer corona discharge distance and longer time.

It was predicted that the AL surface roughness is directly correlated to the amount of sap that flows out through the damaged structure. As such, it is thought that the AL adhesion strength depends on the amount of flowed-out sap. In fact, it is difficult to quantitatively measure the volume of the sap released as a function of corona-discharge. The reasons are as follows: First, although the volume of sap contained in leaves usually depends on the sizes of the leaves, there should be individual differences between the leaves according to the plant’s health. Second, in the attachment process of an AL, it is necessary to press the AL to a certain substrate (Fig. 1). The volume of the released sap might differ in that process. Nevertheless, we can predict the relationship between the volume of the sap released as a function of corona-discharge and the experimental condition of corona-discharge. The ALs were attached to a wall and then pulled with the Instron tensile-force tester to measure the AL adhesive strength (Fig. 5). The corona-treatment conditions (i.e., *d*^*corona*^ and *t*^*corona*^) are the same as those from the surface-roughness analysis. The adhesion-strength data were acquired from the force–distance (F–D) curve. Specifically, at *t*^*corona*^ = 30 s, the F–D curves are not significantly different regardless of the *d*^*corona*^, as can be seen in Fig. 5(a). In contrast, it seems that the F–D curves at *t*^*corona*^ = 60 s are slightly larger than those that at *t*^*corona*^ = 30 s, which is shown in Fig. 5(b). Interestingly, the AL adhesion at *t*^*corona*^ = 60 s is the largest at *d*^*corona*^ = 5 mm. This trend can be clearly seen in the surface plot of Fig. 5(c) that exhibits the relationship between the three factors, the AL adhesion strength, the *d*^*corona*^, and the *t*^*corona*^. We found that the adhesion force of AL increases when the distance between the electrode and the leaf surface gets closer or the corona-treatment time gets longer. Under an assumption that the adhesion force of AL is proportional to the volume of sap, the results represent that the volume of released sap tends to be proportional to the time of corona-treatment and be inversely proportional to the distance between the electrode and the leaf surface.

**Figure 6:**
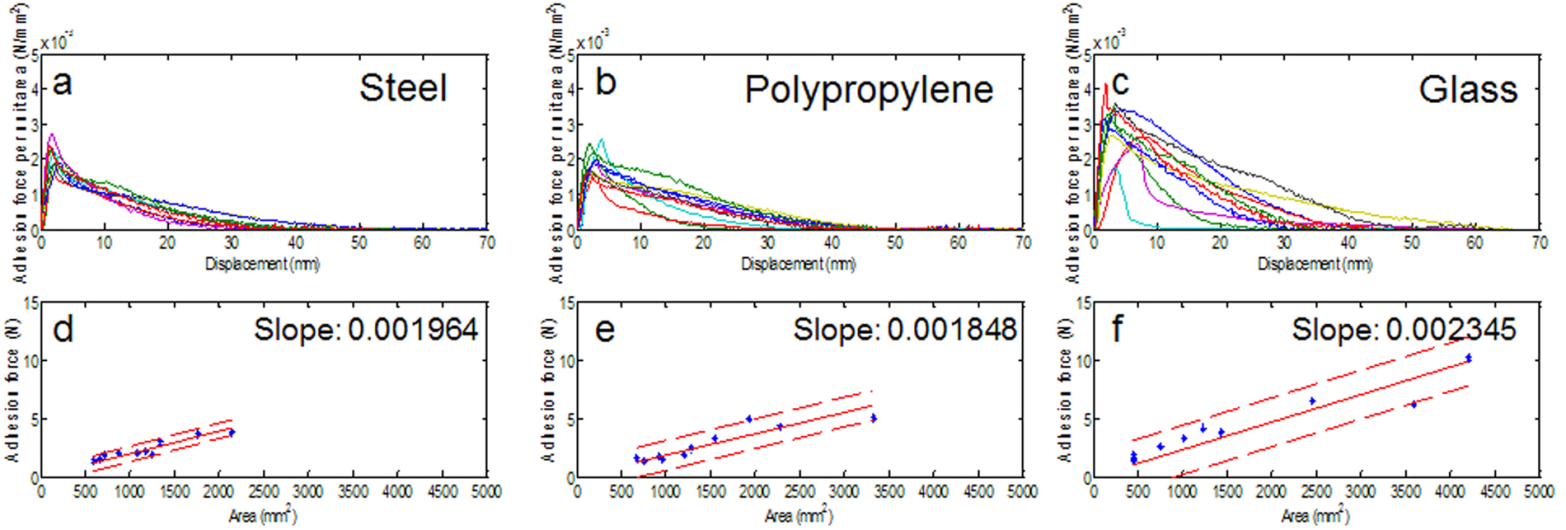
Adhesive forces per unit area-displacement curves obtained on the surface of steel **(a)**, polypropylene **(b)**, and glass **(c)**. **(d–f)** A linear relationship between adhesive force and surface area of AL was found for all substrates. Regardless of the substrate, adhesion forces increase with a larger surface area of AL.

To confirm whether the AL adheres effectively to other materials, experiments were conducted to measure the AL adhesive strength and the adhesive force per single area using polypropylene, glass, and steel (Fig. 6). In all of the experiments, the AL was created with a *d*^*corona*^ of 10 mm. In the condition of *d*^*corona*^ = 5 mm, the corona-discharge often causes too much destruction of the surface of AL. It may be attributed to the high intensity of corona-discharge due to the short distance^15^. As the distance *d*^*corona*^ increases, we confirmed that the phenomenon decreases remarkably. We empirically determined that the *d*^*corona*^ = 10 mm is proper.

Notably, the AL performance could often be varied not only due to the AL size; indeed, in the tests with various substrate materials, the AL ability showed a sensitivity to the spot size, but it is too difficult to control the spot size with a handheld corona-discharge device. As an alternative to the making of the ALs with one corona-treated spot, a corona-discharge treatment was applied to the entire leaf surfaces. In the experiment, the time of corona-treatment depended on the size of the leaves and was usually less than 2~3 min per one AL. The results show that the AL adhesive strength is seemingly higher in the sequential order of glass, polypropylene, and steel. Specifically, both the AL area and adhesion showed the linear characteristics of 1.964, 1.848 and 2.345 mN/mm^2^ for steel, polypropylene, and glass, respectively. We speculate on the reasons for the variations along the linear curve. The fact that every leaf has distinctive vein means each leaf has different sap content and distribution^20,21^. The randomness of poration characteristics could also be the reason. Every corona-treated leaf shows diverse pore structure, location, and number. Nevertheless, the results indicate that the AL that is produced by the corona discharge can be attached to various substrates. Together, Fig. 6 represents two findings: First, the area of the AL is proportional to the adhesion strength. Second, AL can be attached to various types of surfaces. In this experiment, we made AL with leaves from a species of plant (*Heteropanax fragrans*) and measured its adhesion properties on only three types of surfaces. To better understand the adhesion properties of AL, we need a further study using more species of leaf on various substrates.

In this study, to make AL, we have treated corona-discharge only on the upper epidermis of leaves. Because the lower epidermis has protruding structures such as primary and secondary veins, we have thought that the veins could affect the adhesion properties of AL. To compare the effect of corona-discharge treatment on plant leaf surface between upper and lower epidermis, we prepared two kinds of leaves with different structures (a curved leaf: *Dracaena reflexa – Song of Jamaica*; a flat leaf: *Schefflera arboricola*). The distance between the electrode and leaf surface was approximately 10 mm and the time of corona-treatment was 60 s. The curved leaf can be attached to the wall (of a fume hood) after corona-treatment regardless of the external structure of the leaves (Fig. S5). In addition, regardless of whether the leaves were treated front or back (*i.e*., upper or lower epidermis), the sap exudes through the damaged epidermis. What we found was that the attachment duration was shortened when the lower epidermis was treated; it only lasted few seconds (Fig. S5b). We thought that the curved structure might hinder the leaves from stably being attached. We also conducted the same experiment using flat leaves to see if our thoughts are correct (Fig. S5c). As a result, AL stayed well on the wall regardless of whether the leaves were treated front or back. However, the lower epidermis-treated AL could not last more than 10 s (Fig. S5d). We believe that the protruding primary vein structure of the lower epidermis affects the adhesion of AL. To verify our hypothesis, we proceeded the same experiment using leaf fragments (of *Schefflera arboricola*) that do not contain the primary vein (Fig. S5e). As a result, both cases (upper or lower epidermis) showed stable adhesion ability. Taken together, it is fair to say that the adhesion ability of AL is stable regardless of whether the leaves were treated front or back, only if the leaves are flat without any protruding parts like a primary vein.

In conclusion, an investigation of the adhesion of the corona-treated leaves was first conducted, thereby demonstrating the adhesion mechanism. Specifically, the corona discharge can delicately destroy the leaf-surface structure, and the sap flows out through the destroyed structure to form an adhesive force on the leaf surface. This adhesion force is sufficient to withstand the weight of the leaf itself (several milligrams) and results in an attachment to various substrate materials, such as steel, polymer, and glass. The authors believe that an adhesive mechanism that is due to the leakage of a viscous liquid through a micropore surface can be applied in the development of a novel bandage for applications like the treatment of wounds.

## Methods

### Corona-discharge treatment

The BD-10A hand-held corona-discharge device with a 230-V high-frequency generator (Electro-Technic Products, U.S.A.) was used to create the AL. The corona-discharge, brought on by the ionization of air, was performed near the center of the leaves as much as possible to minimize the deviation of the plasma stream from the leaf area (Fig. S1). (Caution: Because the corona discharge produces high voltages and electrical currents, any conductive materials that are around the experimental environments need to be removed.) The distances between the electrode and the leaf surface were kept 5, 10, and 15 mm away and the treatment times for corona-discharge were 30 and 60 s. Immediately after the corona-treatment, ALs can be attached to various substrates such as cement wall, glass, steel, and polymers by a simple clicking motion to the center of AL.

### Thermal imaging of the AL

The corona-treatment-driven temperature changes in the ALs were quantitatively analyzed using thermal imaging and the corresponding FLIR ONE software (FLIR, U.S.A.). After a 30-s corona treatment, time-lapse images of a leaf were immediately acquired for 2 min with a 3-s time interval at room temperature. The leaves were on a sheet of laboratory paper on a table without any fixation.

### SEM imaging of the AL

The morphology and structure of the AL before and after the corona treatment were characterized using the JSM-6701F field-emission scanning electron microscopy (FE-SEM) device (JEOL, Japan). A platinum (Pt)-coating application was conducted for 120 s before the experiment. The SEM was operated at 10 kV, and the imaging spots were randomly selected on the AL surfaces. The SEM micrographs for each spot were collected at different magnifications (×50, ×250, and ×900).

### Optical imaging of the AL

An inverted microscope (AE31E, Shinhan Scientific, Korea) was used to observe the AL surfaces before and after the corona treatment. Calculation of surface roughness (*R*_*q*_) of the leaves is performed using ImageJ (NIH, USA) with SurfCharJ plugin^22^ by the following equation:

$${R}_{q}={(\frac{1}{{N}_{x}{N}_{y}}\sum _{i=1}^{{N}_{x}}\sum _{j=1}^{{N}_{y}}{z}_{ij}^{2})}^{1/2},$$

where *z* is height values of the surface, *N* is the direction of outward surface normal vector, and *x* and *y* are the coordinates in the plane direction. For quantitative analysis of damaged structures of leaf surfaces after corona-treatment, the value of *R*_*q*_ that calculated after corona-treatment was normalized by the value of *R*_*q*_ that calculated before corona-treatment (Fig. 4c).

### FTIR analysis of the AL

The Fourier-transform infrared (FTIR) spectra of the sap were acquired using an FTIR spectrometer (Spectrum 100 FT-IR Spectrometer, Perkin Elmer, USA) at a resolution of 4 cm^−1^. The sap was extracted from the surface of several ALs using the corona discharge and was gathered by applying a gentle squeezing. The final sap volume is approximately 2 ml. The spectra of the sap and the control (i.e., distilled water) were recorded in the range of 500–4000 cm^−1^. The absorbance was plotted by subtracting the measured sap spectrum from the control-sample spectrum.

### Measurement of the AL adhesive force using a tensile-force tester

Immediately after the corona treatment, the AL petiole was mounted in the Model 4502 tensile testing machine (Instron, U.S.A.) to determine the AL adhesive force. Accordingly, the lamina part of the AL was attached to the base surface that is well aligned with the load axis, as can be seen in Fig. S4. An initial load of 1 N was applied by changing the clamp-to-clamp distance at which the initial gauge length was defined as zero. The specimens were stretched at the stretching velocity of 150 mm/min until a failure occurred. The effect of the corona-discharge treatment on the AL performance was tested using a comparison of the AL adhesive forces that were measured on a metal surface depending on the corona-treatment time (*i.e*., 30 and 60 s) and the distances between the tip and the leaves (*i.e*., 5, 10, and 15 mm). In addition, the AL adhesive force was evaluated on different substrates (*i.e*., steel, polypropylene, and glass). The adhesive force was normalized by the AL surface area.

## Electronic supplementary material

Supplementary information
