# Supplementary material for: Adhesive Leaf Created by a Corona Discharge
Source: Sci Rep. 2018 Jan 29;8:1737. doi: 10.1038/s41598-018-19328-8 (PMC5788921; doi:10.1038/s41598-018-19328-8)
Supplement: Supplementary file 1 — Supplementary information [file 41598_2018_19328_MOESM1_ESM.pdf]

Supplementary Information

Adhesive Leaf Created by a Corona Discharge

Wonseok Lee,[1,#] Jongsang Son,[2,3,#] Seonghyun Kim,[4,#] Dongmin Yang,[4] Seungyeop Choi,[1] Rodrigo A. Watanabe,[5] Kyo Seon Hwang,[6] Sang Woo Lee,[1] Gyudo Lee[4,*] and Dae Sung Yoon[4,*]

[1] Department of Biomedical Engineering, Yonsei University, Wonju 26493, South Korea

[2] Shirley Ryan AbilityLab, Chicago, IL 60611, USA

[3] Department of Physical Medicine and Rehabilitation, Northwestern University, Chicago, IL 60611, USA

[4] School of Biomedical Engineering, Korea University, Seoul 02841, South Korea

[5] School of Medicine, University of São Paulo, São Paulo, Brazil

[6] Department of Clinical Pharmacology and Therapeutics, College of Medicine, Kyung Hee University, Seoul 02447, Korea

[#] These authors made an equal contribution.

* Corresponding author: D.S.Y. (dsyoon@korea.ac.kr) and G.L. (lkd0807@korea.ac.kr)

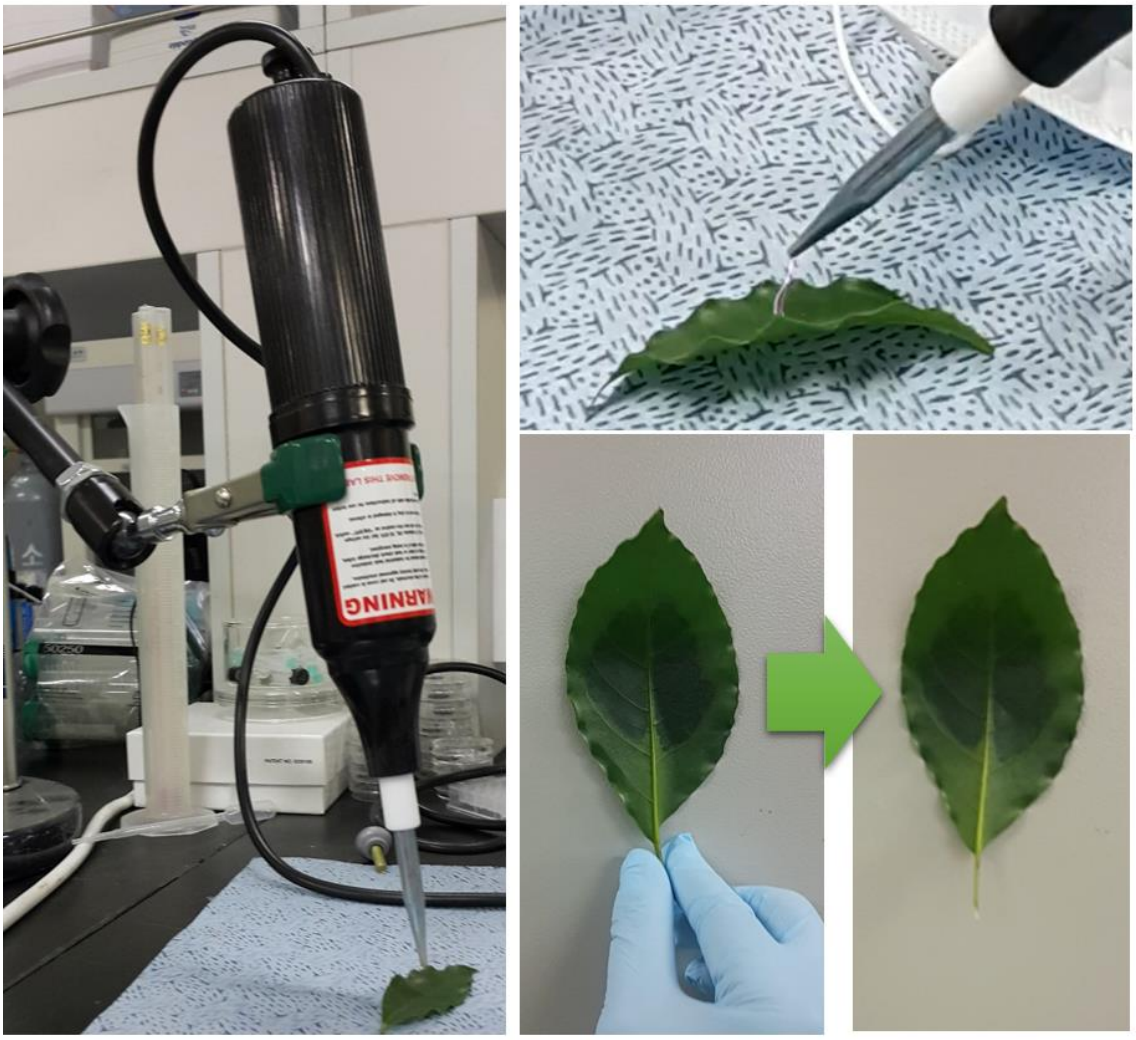

Figure S1. Experimental setup for the fabrication of the adhesive leaf (AL) using a corona discharge.

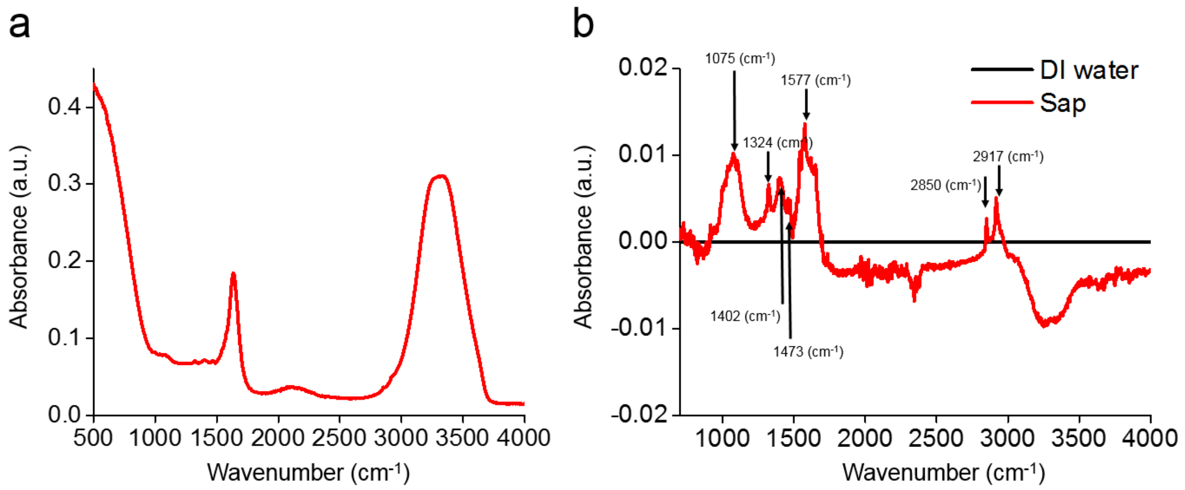

Figure S2. FTIR analysis of the sap taken from the ALs. a) FTIR spectrum of sap (500~4,000 cm^{-1} range). The FTIR spectrum is similar to a glucose solution, indicating that the sap is a glucose-rich solution. b) The filtered spectrum was acquired by subtracting the FTIR spectrum of deionized water from the one of sap.

Fig. S2(a) represents that the FTIR spectrum of sap tends to be similar to that of a glucose solution; the wavenumber is ranged within 1,000~1,100 and 3,000~3,400 cm^{-1} (ref. [1]). That is why we mentioned glucose as a main constituent of sap in the manuscript. The FTIR spectrum of sap would be composed of multiple bands caused by vibrations of various groups belonging to cellulose, hemicellulose and other molecules such as proteins, lipids, carbohydrates, and wax. Specifically, the bands near 1,075 cm^{-1} exhibit skeletal vibrations C-O caused by cellulose [2]. The bands 1,402 and 1,473 cm^{-1} exhibit CH bending and O–CH_3 stretching caused by hemicellulose [2]. The bands between 2,800 and 3,000 cm^{-1} exhibit C–H stretching vibrations caused by lipids and the bands between 1,500 and 1,700 cm^{-1} represent amide I and II bands caused by proteins [3]. Carbohydrates absorption band including amide III bands that mainly located between 1,000 and 1,500 cm^{-1} (Ref. [3]). The vibrations located at 2,918 cm^{-1} are attributed to –CH_2 vibrations and could originate from the presence of wax substances present on the surface of the cell wall [4]. Although we have assumed that such spectra for various components are superimposed on a single spectrum of sap we observed, it is hard to discriminate individual components in the present form of FTIR spectrum of sap. So, we subtracted the FTIR spectrum of deionized water from the one of sap, representing a more filtered spectrum of

sap (Fig. S2b). On the filtered spectrum, we marked the wavelength for each component that we searched.

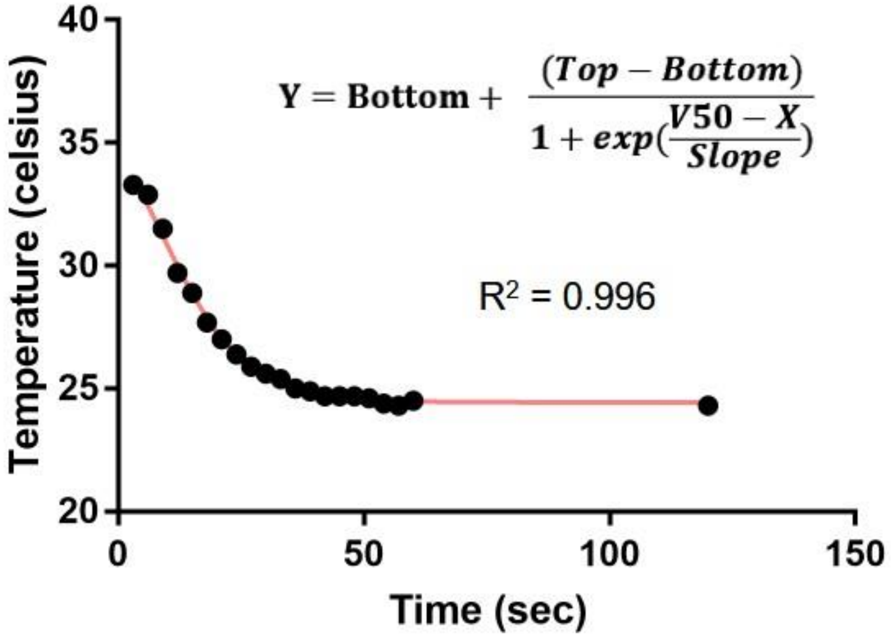

Figure S3. Curve-fitting of the AL temperature variation after a corona discharge. The Boltzmann sigmoid fit was used (Bottom: 38.52; Top: 24.45; V50: 8.387; and Slop: 8.548).

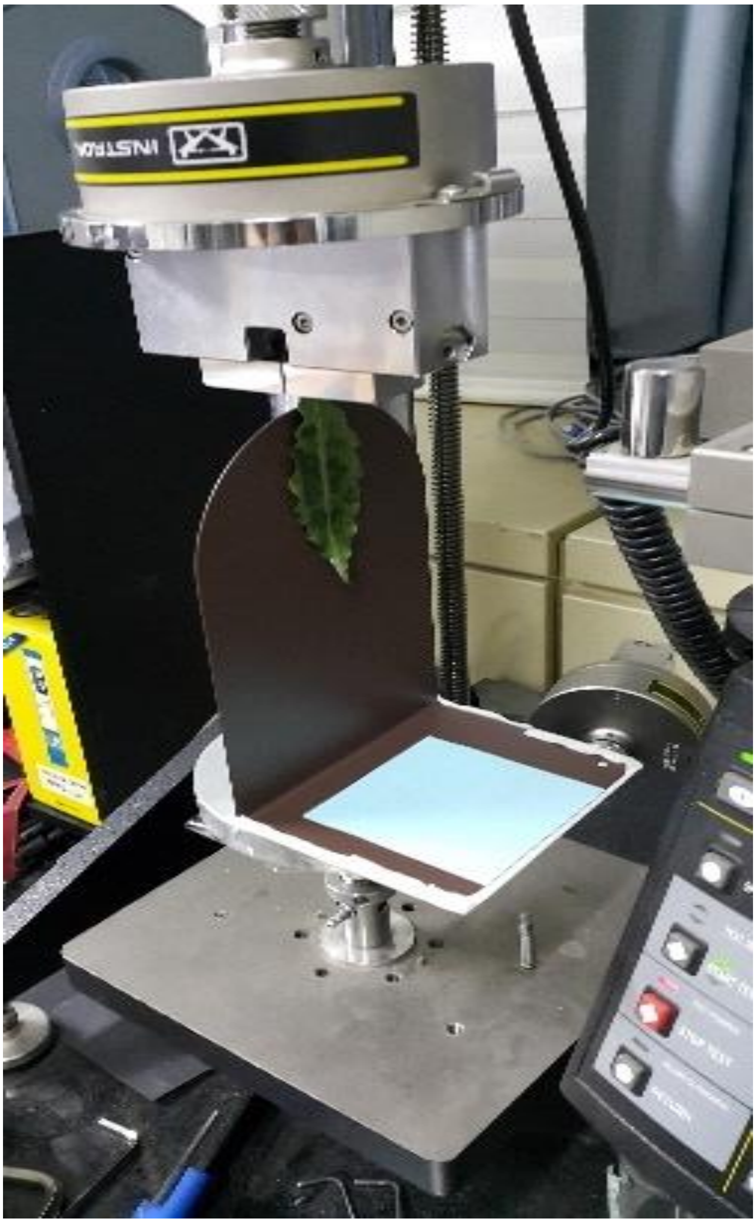

Figure S4. Experimental setup for the AL adhesion-force measurement using an Instron device.

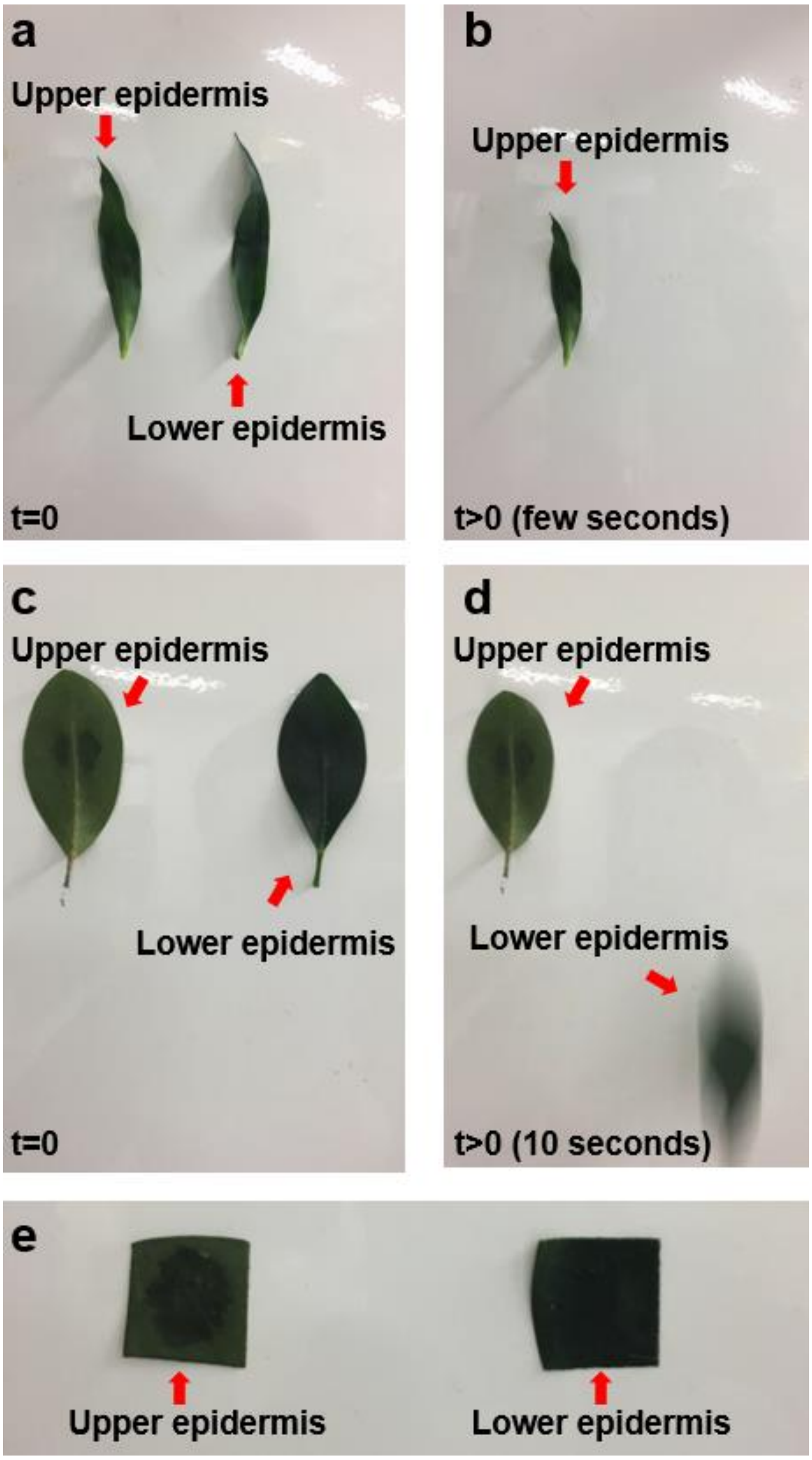

Figure S5. Comparison of adhesion of AL on the wall of a fume hood. a and b) Adhesion of AL using curved leaves (*Dracaena reflexa – Song of Jamaica*). c and d) Adhesion of AL using flat leaves (*Schefflera arboricola*). e) Adhesion of AL fragments (approximately 1.5 × 1.5 cm^2) without the primary vein (*Schefflera arboricola*). In the figure, the marks of "upper epidermis" and "lower epidermis" indicate that the upper and lower epidermis were treated by corona discharge, respectively.

References

1 Li, Y. *et al.* Carbon nanoparticles/soy protein isolate bio-films with excellent mechanical and water barrier properties. *Industrial Crops and Products* **82**, 133-140, doi:https://doi.org/10.1016/j.indcrop.2015.11.072 (2016).

2 Yang, H., Yan, R., Chen, H., Lee, D. H. & Zheng, C. Characteristics of hemicellulose, cellulose and lignin pyrolysis. *Fuel* **86**, 1781-1788, doi:https://doi.org/10.1016/j.fuel.2006.12.013 (2007).

3 Wei, Z., Jiao, D. & Xu, J. Using Fourier Transform Infrared Spectroscopy to Study Effects of Magnetic Field Treatment on Wheat (Triticum aestivum L.) Seedlings. *Journal of Spectroscopy* **2015**, 6, doi:10.1155/2015/570190 (2015).

4 Abidi, N., Hequet, E. & Cabrales, L. (2011). Applications of Fourier Transform Infrared Spectroscopy to Study Cotton Fibers, Fourier Transforms - New Analytical Approaches and FTIR Strategies, Prof. Goran Nikolic (Ed.), InTech, DOI: 10.5772/15829. Available from: https://www.intechopen.com/books/fourier-transforms-new-analytical-approaches-and-ftir-strategies/applications-of-fourier-transform-infrared-spectroscopy-to-study-cotton-fibers.
